# Cortical activation during auditory working memory varies with hearing aid use status in age-related hearing loss

**DOI:** 10.1038/s41598-026-59089-3

**Published:** 2026-06-23

**Authors:** Allison S. Hancock, Naveen K. Nagaraj, Mindee L. Anderson, Alan Wisler, Tiffany Shelton, Bridger L. Jorgensen, Ronald B. Gillam

**Affiliations:** 1 Alzheimer’s Disease and Dementia Research Center, Utah State University, Logan Utah, USA; 2 Patient Services Research, Cincinnati Children’s Hospital Medical Center, Cincinnati, OH USA; 3 Department of Pediatrics, University of Cincinnati College of Medicine, Cincinnati, OH USA; 4 Department of Speech and Hearing Sciences, Utah State University, Logan Utah, USA; 5 Department of Mathematics and Statistics, Utah State University, Logan Utah, USA

**Keywords:** Age-related hearing loss, Hearing aids, fNIRS, Working memory, Listening Effort, N-Back, Neuroscience, Psychology, Psychology

## Abstract

Age-related hearing loss (ARHL) is associated with increased listening effort due to greater cognitive demands needed for supporting effective communication. Verbal working memory (WM) is an important mechanism supporting speech understanding in adults with ARHL. Using functional near-infrared spectroscopy and an auditory N-back task (0-, 1-, 2-back load), we examined whether hearing aid status was associated with cortical activation in adults with ARHL. Three groups were compared: typical-hearing adults, experienced hearing aid users, and newly fitted users. Linear mixed-effects models revealed significant Group × Load interactions across the left superior temporal gyrus (STG), left inferior parietal lobule (IPL), and left dorsolateral prefrontal cortex (DLPFC). Experienced users exhibited accuracy comparable to the typical-hearing adults and showed STG and IPL activation patterns more similar to the typical-hearing group, whereas newly fitted users showed reduced activation under higher WM load. These findings indicate that cortical activity during an auditory N-back task engaging verbal WM differs as a function of hearing aid status and is not explained by aided audibility alone. Overall, experienced users exhibited load-dependent cortical activation patterns that more closely resembled those of typical-hearing adults than newly fitted users during an auditory N-back task.

**Supplementary Information:**

The online version contains supplementary material available at https://doi.org/10.1038/s41598-026-59089-3.

## Introduction

Age-related hearing loss (ARHL) is among the most prevalent chronic conditions in older adulthood and is associated with poorer health outcomes, including increased risk of depression and social isolation. ARHL has also been identified as a potentially modifiable risk factor for dementia^1–4^. As auditory input becomes degraded due to hearing loss, listeners increasingly rely on working memory (WM) resources to understand spoken language during ongoing conversations^5^. Although hearing aids improve speech audibility, many adults with ARHL continue to report difficulty understanding speech in noise^6,7^, consistent with the idea that successful speech understanding depends on more than improvements in auditory thresholds alone^8^. Further, verbal WM capacity has been associated with degraded speech perception in both young and older adults, suggesting WM plays an important role in speech processing^9,10^. However, it remains unclear whether hearing aid experience is associated with differences in cortical activation supporting verbal WM. Addressing this question is important given the high prevalence of untreated hearing loss, which affects nearly half of adults over the age 75 ^3^. Neurophysiological evidence is therefore needed to clarify how hearing-aid experience may relate to cortical activation supporting verbal WM in adults with hearing loss.

Several theoretical accounts have suggested that hearing loss increases reliance on WM and attention^5–7^. The Information Degradation Hypothesis proposes that hearing loss increases the cognitive processing demands required to understand speech^11^. Under this account, cognitive resources are diverted toward perceptual processing, leaving fewer resources available for other tasks, such as WM updating, attention, and comprehension. In contrast, the Framework for Understanding Effortful Listening (FUEL) conceptualizes listening effort as the allocation of limited cognitive resources in response to the interaction between task demands, listener capacity, and motivational factors^6^. Whereas the Information Degradation Hypothesis emphasizes the bottom‑up consequences of degraded input, FUEL highlights top‑down regulation of effort in relation to speech perception and available cognitive resources. These frameworks provide somewhat different mechanistic rationales for why individuals with hearing loss show altered patterns of cortical activation during demanding verbal WM tasks.

Based on the Ease of Language Understanding model, WM has been shown to support language understanding under degraded listening conditions^5,9^. When incoming speech input does not readily match stored phonological representations, explicit WM resources are engaged to resolve this mismatch during ongoing linguistic processing^5^. The N-back task provides a well-established paradigm for measuring WM processes, as it requires participants to store, manipulate, and update items while inhibiting irrelevant information^12^. During the N-back, participants must identify whether the current stimulus matches the stimulus presented “N” trials before, allowing for systematic manipulation of cognitive load^13^. N-back paradigms can include a 0-back condition, in which participants indicate each time they hear a predefined stimulus. The 0-back task requires sustained attention but has minimal storage or retrieval demands; therefore, it is often used as a covariate to statistically control for sustained attention, allowing analyses to focus on storage, recall, inhibition, and updating components of WM ^14,15^.

Neuroimaging studies using N-back tasks consistently implicate a fronto-parietal network in WM ^16–18^, including the inferior parietal lobule (IPL), which supports encoding and phonological storage, and the left dorsolateral prefrontal cortex (DLPFC), which mediates monitoring, filtering, and executive control^19,20^. Given the auditory N-Back task used in this study, these processes are left-lateralized in right-handed individuals, particularly within the frontoparietal regions supporting phonological processing and executive control^18,19^. These regions also interact with the superior temporal gyrus (STG) to support acoustic and phonological processing, forming a functional network^8,21^. Currently, limited neurophysiological evidence has examined how hearing aid experience relates to cortical activation patterns within this network during increasing WM demands. Measuring cortical activation during tasks that specifically tax WM, such as the N-back, provides an experimental approach for assessing whether hearing aid experience is associated with load-related changes in activation across temporal and fronto-parietal regions.

Functional near-infrared spectroscopy (fNIRS) has been increasingly used to study cortical activation because it provides a complementary approach to fMRI. Although specialized fMRI approaches (e.g., sparse imaging) can reduce scanner noise^23^, fNIRS operates quietly, is tolerant of movement, and is compatible with hearing devices^21^. However, fNIRS has important limitations, including limited depth penetration and lower spatial resolution relative to fMRI^24^. Despite these constraints, fNIRS has been successfully used to examine cortical activation during speech perception and listening effort tasks, particularly in populations using hearing devices^25–28^.

Rovetti and colleagues^27^ found that prefrontal oxygenation increased with WM load during both auditory and visual N-back tasks in older adults with hearing loss. In a within-subject comparison of experienced hearing aid users, hearing aids did not significantly reduce group-level prefrontal oxygenation during the auditory N-back task, nor did they improve behavioral measures such as self-reported effort, reaction time, or percent correct. These results suggest that amplification alone did not substantially alter neural or behavioral indices at the group level. Because the study design compared aided and unaided performance within experienced users, it primarily assessed the immediate effects of amplification rather than differences related to hearing aid experience. Furthermore, the absence of a typical hearing control group limited the ability to interpret cortical activation patterns in experienced users compared to adults without hearing loss. Prior fNIRS work further shows that increasing N-back load is associated with greater recruitment of prefrontal regions, including DLPFC, across auditory and visual modalities, supporting its role in domain-general WM processing^25^. To our knowledge, no fNIRS studies have examined newly fitted hearing aid users as a proxy for the pre-acclimatization stage while simultaneously comparing them with experienced users and typical-hearing adults.

## Current study

The present study tested how hearing-aid use relates to cortical activation of auditory and WM networks during an auditory N‑back task. Using a cross-sectional design, we compared newly fitted hearing aid users with ARHL who had no prior hearing aid experience (new users), experienced hearing aid users with at least 6 months of device use (experienced users), and typical-hearing adults without age-related hearing loss (typical-hearing) to examine whether hearing aid use status is associated with differences in load‑dependent cortical activation. The auditory N-back task presented spoken digits 0-, 1-, and 2-back conditions that systematically varied WM load. We hypothesized that experienced users would exhibit patterns of activation in auditory (left STG) and WM related regions (left IPL, left DLPFC) that more closely resemble those observed in typical-hearing adults as task demands on WM increased. In contrast, new users, assessed shortly after initial fitting, were expected to show reduced STG activation and greater prefrontal activation (left DLPFC) under high WM load. Examining these neurophysiological differences across groups has the potential to provide insight into how hearing aid experience influences neural systems supporting verbal WM.

## Results

Pairwise relationships among descriptive variables are displayed in Table 1. Statistical significance was evaluated using a Bonferroni-corrected threshold (α = 0.0033). N-Back accuracy was significantly correlated across task conditions, with 0-back accuracy correlated with 1-back (*r* = .46, *p* < .001) and 2-back (*r* = .46, *p* < .001), and 1-back accuracy correlated with 2-back (*r* = .59, *p* < .001). Age was negatively associated with 2-back accuracy (*r* = –.48, *p* < .001). Neither audibility, indexed by the Speech Intelligibility Index (SII), nor global cognition, measured using the Montreal Cognitive Assessment (MoCA), was significantly correlated with N-back accuracy.

**Table 1:** Pearson correlations among age, SII, MoCA, and N-back accuracy.

Variable	Age	SII	MOCA	ACC 0-back	ACC 1-back	ACC 2-back
Age	–	− 0.35	− 0.25	− 0.25	− 0.34	− 0.48*
SII	− 0.35	–	0.14	0.26	0.30	0.26
MOCA	− 0.25	0.14	–	0.22	0.15	0.25
ACC 0-back	− 0.25	0.26	0.22	–	0.46*	0.46*
ACC 1-back	− 0.34	0.30	0.15	0.46*	–	0.59*
ACC 2-back	− 0.48*	0.26	0.25	0.46*	0.59*	–

Values represent Pearson correlation coefficients. Statistical significance was evaluated using a Bonferroni-corrected threshold of α = 0.0033 (0.05/15). Significant correlations are indicated by *p < .0033. SII= Speech Intelligibility Index; MoCA = Montreal Cognitive Assessment; ACC= accuracy.

### N-back accuracy

**Fig. 1:**
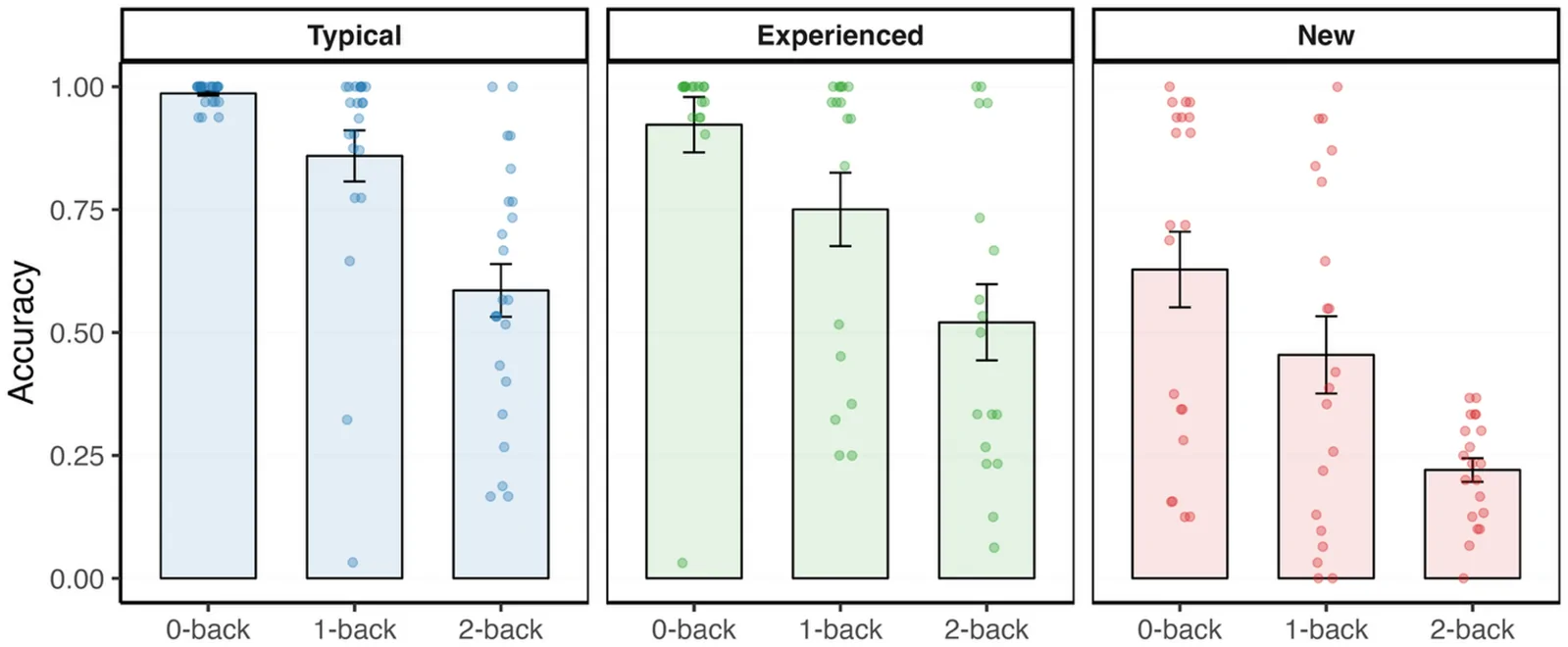
Mean accuracy across N-back task conditions by group. Bars show mean accuracy by task and group; error bars represent SEM, and points indicate individual participant mean values. Typical = typical-hearing adults (blue); Experienced = experienced hearing aid users (green); New = new hearing aid users (red).

N-back response accuracy was assessed using a mixed-effects logistic regression model with random intercepts for participant and trial. Accuracy declined significantly with increasing task difficulty, with lower performance on the 1-back (b = − 2.69, *p* < .001) and 2-back (b = − 4.52, *p* < .001) conditions relative to the 0-back baseline. Age was also associated with reduced accuracy (b = − 0.07, *p* = .003). A likelihood-ratio test revealed that including the Task × Group interaction significantly improved model fit (χ²(4) = 50.63, *p* < .001), indicating that the effect of WM load differed across groups. Post hoc comparisons showed that typical-hearing adults and experienced users performed similarly on the 1-back and 2-back conditions. In contrast, new users showed significantly lower accuracy than typical-hearing adults and experienced users at higher WM loads (all *p*s ≤ 0.001). Descriptive accuracy values and standardized effect sizes for each task condition are presented in Table 2, and mean accuracy (± SEM) by group and task is visualized in Fig. 1. Full model estimates are provided in Supplementary Table S1.

**Table 2:** Mean N-back accuracy and between-group effect sizes.

Task	Typical-hearing (*n* = 23)	Experienced users (*n* = 17)	New users (*n* = 20)	Hedges’ g (Typical vs. Experienced)	Hedges’ g (Typical vs. New)	Hedges’ g (Experienced vs. New)
0-back	0.99 (0.02)	0.92 (0.23)	0.63 (0.34)	*g* = 0.38	*g* = 1.44	*g* = 0.98
1-back	0.86 (0.24)	0.75 (0.31)	0.45 (0.35)	*g* = 0.38	*g* = 1.31	*g* = 0.88
2-back	0.59 (0.26)	0.52 (0.32)	0.22 (0.12)	*g* = 0.22	*g* = 1.82	*g* = 1.24

Values are mean (SD) accuracy by task and group. Hedges’ g = standardized mean difference.

### FNIRS results

**Fig. 2:**
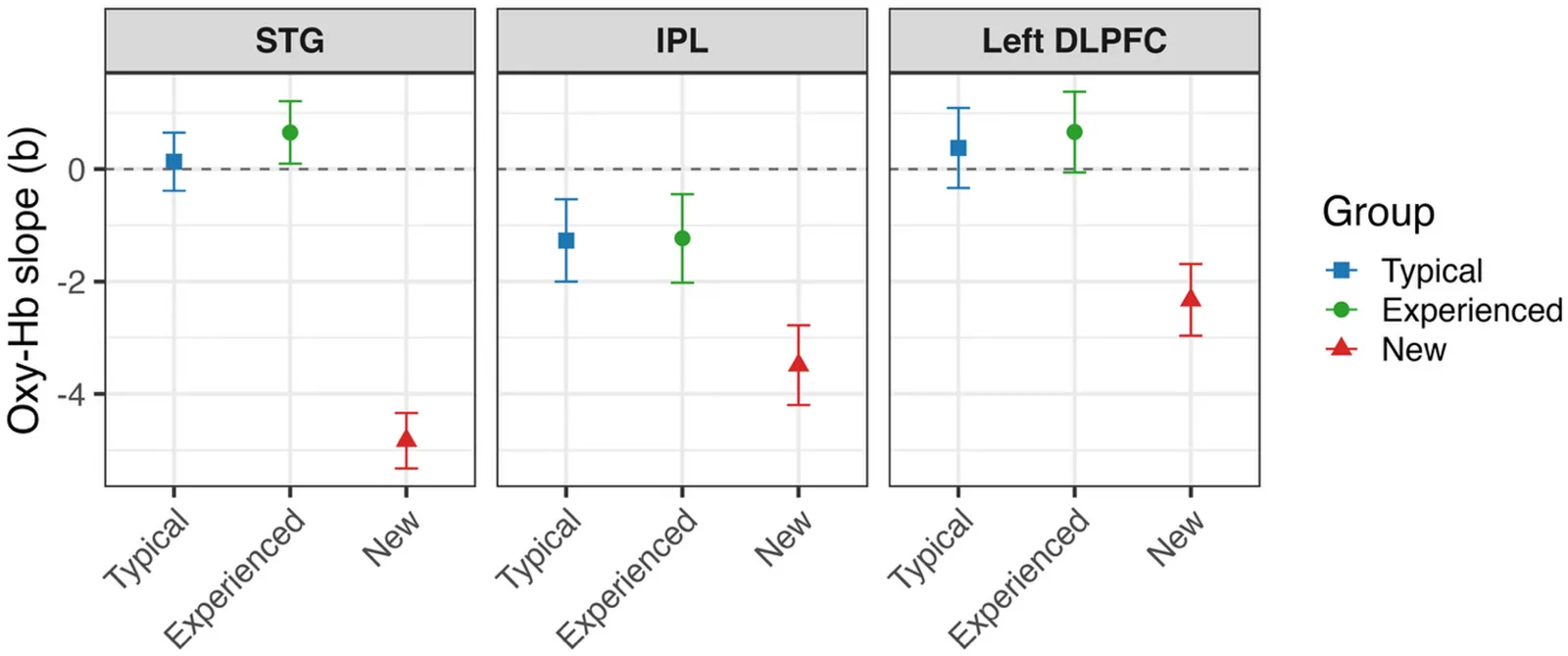
Simple slope estimates of load-related changes in Oxy-Hb activation by group and region of interest. Points represent model-derived simple slope estimates describing the change in Oxy-Hb activation from 1-back to 2-back for typical-hearing adults (Typical), experienced hearing-aid users (Experienced), and new hearing-aid users (New) within the left superior temporal gyrus (STG), left inferior parietal lobule (IPL), and left dorsolateral prefrontal cortex (left DLPFC). Error bars represent 95% confidence intervals. Negative values indicate declining activation, whereas positive values indicate higher (or less negative) activation at 2-back relative to 1-back.

**Fig. 3:**
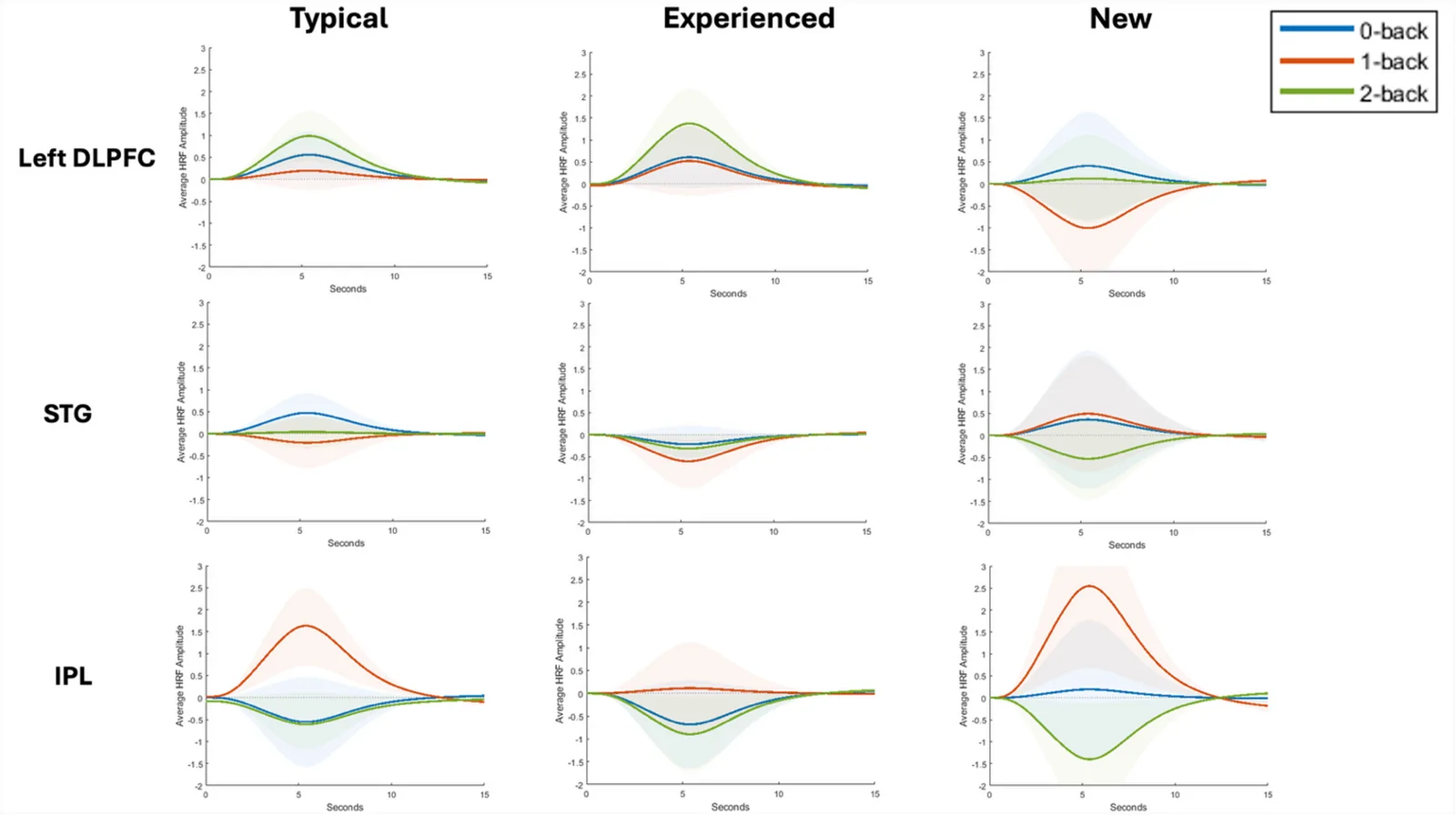
Group-level Oxy-Hb hemodynamic response functions by task and region of interest. Group-averaged Oxy-Hb hemodynamic response functions for 0-back, 1-back, and 2-back conditions in the left superior temporal gyrus (STG), left inferior parietal lobule (IPL), and left dorsolateral prefrontal cortex (left DLPFC) for typical-hearing adults (Typical), experienced hearing-aid users (Experienced), and new hearing-aid users (New). Shaded regions represent ± 1 SE.

Mixed-effects regression models assessed the effects of Group (typical-hearing, experienced users, new users) and Load (1-back, 2-back) on oxygenated hemoglobin (Oxy-Hb) beta values within STG, IPL, and left DLPFC regions, while controlling for 0-back activation, SII, and accuracy (Table 3). Significant Group × Load interactions were followed by simple slopes analyses to examine load-dependent changes in activation from 1-back to 2-back. The model-estimated load-related changes are summarized in Fig. 2. Full simple slopes and pairwise comparisons for each region are provided in Supplementary Tables S2–S4. To facilitate interpretation of the Group × Load interactions, mean ROI Oxy-Hb hemodynamic response (HRF) plots are shown in Fig. 3. Because the mixed-effect models estimate conditional load effects, these plots are intended to illustrate the responses underlying the interaction effects rather than directly reproduce model coefficients.

### STG

A significant Group × Load interaction was observed for Oxy-Hb beta values in the STG, *F*(2, 19243) = 138.97, *p* < .001, indicating that load-related changes in activation differed across groups (Table 3). To interpret this interaction, we calculated simple slopes describing the change in activation from 1-back to 2-back within each group and their corresponding confidence intervals. Typical-hearing adults showed minimal change in activation between 1-back and 2-back conditions (simple slope estimate = 0.13, 95% CI [− 0.38, 0.65]), whereas experienced users showed a modest increase in activation (simple slope estimate = 0.65, 95% CI [0.10, 1.21]). In contrast, new users showed a marked decline in activation as task load increased (simple slope estimate = − 4.83, 95% CI [− 5.33, − 4.34]; Fig. 1). Pairwise comparisons confirmed that this load-related decline for new users differed significantly from both typical-hearing and experienced users (*ps* < 0.001) whereas no significant difference was detected in the slopes for typical-hearing and experienced users (*p* = .36). The HRF plots (Fig. 3) show comparatively small changes in activation from 1-back to 2-back in typical-hearing adults and experienced users, whereas new users show a marked decline in activation at 2-back.

### IPL

Oxy-Hb beta values in the IPL also showed a significant load-related difference across groups (Group × Load interaction), *F*(2, 9519.9) = 12.43, *p* < .001(Table 3). Simple slopes indicated that activation decreased from the 1-back to the 2-back condition for all groups, but the magnitude of this decline differed across groups. Typical-hearing adults showed a moderate reduction in activation with increasing task load (simple slope estimate = − 1.27, 95% CI [− 2.00, − 0.54]), and experienced users exhibited a similar pattern (simple slope estimate = − 1.23, 95% CI [− 2.02, − 0.45]). In contrast, new users showed a substantially larger decline in activation as task load increased (simple slope estimate = − 3.49, 95% CI [− 4.20, − 2.78]; Fig. 2). Pairwise comparisons confirmed that this load-related decline for new users differed significantly from both typical-hearing and experienced users (*ps* ≤ 0.001), whereas the slopes for typical-hearing and experienced users did not show a statistically significant difference (*p* = .99). The HRF plots (Fig. 3) further illustrate the inverted-U pattern across load, with a more pronounced decline from 1-back to 2-back in new users.

### Left DLPFC

A similar pattern was observed in the left dorsolateral prefrontal cortex (DLPFC), where Oxy-Hb beta values differed across groups as a function of task load (Group × Load interaction), *F*(2, 9464.3) = 24.48, *p* < .001 (Table 3). Simple slopes indicated that activation showed little change between the 1-back and 2-back conditions for typical-hearing (simple slope estimate = 0.38, 95% CI [− 0.33, 1.09]) and experienced users (simple slope estimate = 0.66, 95% CI [− 0.06, 1.38]). In contrast, new users exhibited a marked decline in activation with increasing task load (simple slope estimate = − 2.33, 95% CI [− 2.97, − 1.69]; Fig. 2). Pairwise comparisons again indicated that the slope for new users differed significantly from both typical-hearing and experienced users (*ps* < 0.001), whereas no statistically significant differences in the slopes of typical-hearing and experienced users were found (*p* = .85). The HRF plots (Fig. 3) show a load-dependent increase in activation, with larger responses at 2-back relative to 0 and 1-back loads in typical-hearing adults and experienced users. In contrast, new users show a peaked response at 1-back, rather than the continued increase observed in the other groups.

**Table 3:** Summary of results for mixed-effects regression models predicting Oxy-Hb across regions of interest.

Term	STG		IPL		Left-DLPFC	
	Estimate	* p*-value	Estimate	* p*-value	Estimate	* p*-value
Intercept	− 8.062	0.212	− 9.341	0.157	− 4.761	0.474
Experienced users	3.561	0.288	4.650	0.176	4.193	0.229
New user	4.184	0.113	3.690	0.170	4.301	0.116
2-back	0.133	0.613	**− 1.269**	**0.001**	0.379	0.296
SII	0.099	0.203	0.127	0.112	0.040	0.614
ACC	0.287	0.127	**−** 0.322	0.231	**−** 0.517	0.038
0-back Oxy-Hb	**− 0.121**	$\:<\:$ **0.001**	**− 0.094**	$\:<\:$ **0.001**	**− 0.030**	**0.008**
Experienced: 2-back	0.520	0.172	0.037	0.946	0.283	0.578
New users: 2-back	**− 4.964**	$\:<\:$ **0.001**	**− 2.221**	$\:<\:$ **0.001**	**− 2.709**	$\:<\:$ **0.001**

Model Fit						
AIC	145413.052	72362.826	70558.667			
BIC	145507.468	72448.830	70644.608			
Log-likelihood	− 72694.526	− 36169.413	− 35267.333			

STG = superior temporal gyrus; IPL = inferior parietal lobule; left DLPFC = left dorsolateral prefrontal cortex; SII = Speech Intelligibility Index; ACC = accuracy. Coefficient estimates and *p*-values are reported for all fixed effects, along with model fit indices (AIC, Akaike information criterion; BIC, Bayesian information criterion; log-likelihood). Each model included random intercepts for participant and channel. A Bonferroni-corrected significance threshold of *p* < .0167 was applied across the three ROI models. Values meeting the corrected threshold are shown in bold.

## Discussion

Our findings identified neurophysiological group-level differences in load dependent cortical activation during an auditory N-back task associated with hearing aid use status. Specifically, new users showed greater reduction in cortical activation from 1-back to 2-back across the left STG, left IPL, and left DLPFC, whereas experienced users exhibited activation patterns that were more similar to those observed in typical-hearing adults. Although these differences are associated with hearing aid use status, the cross‑sectional design precludes the determination of whether they reflect hearing aid experience dependent neural adaptation or pre‑existing individual differences.

While causal inference cannot be drawn, the observed response patterns can be interpreted with the Information Degradation Hypothesis, ELU, and the FUEL frameworks^5,6,8^. Specifically, the reduced or more negative Oxy-Hb observed in new users may reflect reduced neural efficiency or limited compensatory capacity under increased listening and WM demands, potentially restricting the resources available for updating and refreshing digits in the WM. Within the FUEL framework, the observed divergence in cortical activation from 1-back to 2-back across groups may reflect differences in how cognitive resources were allocated as task demands increased. Specifically, typical-hearing adults and experienced users maintained or slightly increased activation from 1-back to 2-back, suggesting sustained engagement of task-relevant networks under increasing WM load. In contrast, new users showed reduced activation and performance under higher task demands, which may indicate that task demands exceeded available cognitive resources, resulting in reduced engagement. Because the N-back task relies on closed set digit recognition with increasing WM load, the observed group differences in load-dependent cortical activation are more likely to reflect differences in WM demands across groups rather than auditory processing difficulties per se.

The observation that experienced users showed load‑dependent activation patterns resembling those of typical‑hearing adults should not be interpreted as evidence of normalization or optimal processing. Rather, group similarity likely reflects convergence in the form of task‑related activation under increasing WM demand. Such convergence may arise from multiple mechanisms, including long‑term experience managing degraded auditory input with amplification^29,30^, differences in cognitive reserve or task strategy^31^, or selection bias whereby individuals with more resilient neural systems are more likely to adopt and maintain hearing‑aid use. Distinguishing among these possibilities will require longitudinal designs. Importantly, experienced users showed a cortical activation pattern similar to that of typical-hearing adults despite lower aided audibility (SII scores) than new users, suggesting that factors beyond audibility alone contribute to load dependent WM engagement. This interpretation is further supported by the auditory N-back task itself, which places substantial demands on domain‑general WM updating^17^ and relies minimally on speech perception. Performance is unlikely to be constrained by audibility once the digits are clearly audible. One possible interpretation is that hearing aid experience is associated with differences in how WM resources are engaged under increasing task demand, potentially reflecting more efficient or consistent engagement of WM related cortical networks. Alternatively, these group differences may reflect learned strategies for allocating cognitive resources during auditory based tasks^31^, that are not directly attributable to differences in auditory access.

In contrast to experienced users and typical-hearing adults, new users exhibited reduced cortical activation as WM demands increased. Although we hypothesized that new users would show increased activation in left DLPFC, activation in STG, left DLPFC, and IPL instead declined from 1-back to 2-back (Table 3; Fig. 2). The reduction in activation, accompanied by a concurrent decrease in behavioral accuracy, suggests a reduced ability to sustain engagement of auditory and WM regions under increasing task demands. This pattern may reflect altered cortical activation associated with prolonged auditory deprivation^29,32^, particularly given that new users had lived with untreated hearing loss for an average of 11.5 years (range = 1–50 years) prior to amplification. Although we confirmed that the digits were audibly clear to all participants, new users had no opportunity to acclimatize to amplified input as they were tested immediately after receiving their hearing aids. As such, the observed pattern may reflect not only differences associated with auditory experience, but also factors related to device unfamiliarity, task novelty with amplification, or increased attentional demands associated with a new sensory input. These factors may have influenced performance and cortical activation independently of long-term auditory deprivation and therefore, should be considered when interpreting the present findings.

The STG findings provide additional insight into the limits of immediate aided audibility. Although new users were tested with hearing aids, they showed a marked decline in activation in the STG during the 2-back task. Given the role of STG in phonological processing^33^, this reduction may reflect increased demands on verbal WM or reduced engagement of phonological processing as task demands increase, rather than difficulty with encoding of the stimuli^8^. Furthermore, these findings are unlikely to be explained by audibility alone. The N-back task used familiar digits with minimal linguistic complexity, and no correlation was observed between audibility (SII) and N-back accuracy (Table 1), suggesting that adequate perception of the stimuli was achieved. Instead, the reduced STG activation observed in new users may reflect differences in WM updating and attentional processes under increasing task demands.

Taken together, our findings indicate that hearing aid experience is associated with differences in cortical activation during a verbal WM task. Specifically, experienced users demonstrated consistent activation across STG, IPL, and left DLPFC as WM demands increased, with patterns more similar to those observed in typical-hearing adults than to individuals with ARHL but minimal experience wearing hearing aids. Because the study is cross‑sectional, we cannot determine whether these patterns reflect neuroplastic change induced by long‑term hearing‑aid use or pre‑existing individual differences. Nevertheless, the pattern of WM network engagement in experienced users suggests that long‑term experience managing everyday listening demands with amplification may support more robust or efficient engagement of domain‑general cognitive systems during the N‑back task. Future longitudinal studies are needed to clarify whether extended hearing‑aid use contributes to neural adaptation or whether individuals with more resilient cognitive networks are more likely to adopt and consistently use amplification.

## Limitations and future directions

Several limitations should be acknowledged. First, experienced users adopted amplification shortly after noticing hearing difficulties, whereas new users lived with untreated loss for approximately 11.5 years despite similar audiometric profiles. This difference may reflect pre-existing variability in health-seeking behavior, cognitive engagement, motivation, or socioeconomic factors, any of which could contribute to the observed group differences. It is also possible that individuals who successfully adopt and maintain long-term hearing aid use differ in underlying neural or behavioral characteristics prior to intervention. Second, these findings should be interpreted in light of the cross-sectional design, which does not allow determination of whether group differences in neural activation developed with hearing-aid experience or were present before intervention. Future longitudinal work is needed to clarify the direction of this relationship. Additionally, although all participants met the standard MoCA cutoff (≥ 26), this threshold may not fully capture cognitive differences across groups^34^. Third, although newly fitted users provide a comparison group prior to acclimatization, they were tested immediately following hearing-aid fitting, and performance may have been influenced by device unfamiliarity or task novelty with amplification. Further, participants in both hearing-aid groups used different hearing aid devices, which vary in signal processing and noise management features, and may have contributed to variability in cortical activation^28^. Fourth, the primary effects were observed as Group × Load interactions, reflecting WM load-dependent changes in cortical activation rather than differences at a single task level. While this approach may provide a sensitive index of WM related processing, it limits direct interpretation of group differences within individual task conditions. Finally, including the 0-back condition as a covariate was intended to equate groups on sustained attention and reduce baseline differences; however, 0-back activation may also reflect group-related variance associated with hearing status or task engagement, and covarying it may have influenced the magnitude or interpretation of the observed effects. Although no significant differences were observed between typical-hearing adults and experienced users, this does not imply that activation levels are identical. Differences may be present but too small to detect with the current sample size or may be more localized than can be captured by the aggregated regions examined in this study. Future work could consider the use of supervised learning algorithms with embedded variable selection processes to identify relevant regions of activation and to more precisely characterize the differences in activation across groups.

## Conclusions

This study shows that cortical activation patterns during an auditory WM task differs as a function of hearing aid experience. Typical-hearing adults and experienced users exhibited consistent activation across auditory and fronto-parietal regions as WM demands increased, whereas new users showed reduced activation under high WM load, particularly in the STG. These findings are consistent with the Information Degradation Hypothesis, the ELU model, and the FUEL framework, suggesting that hearing aid experience is associated with differences in load-dependent cortical activation during demanding listening based cognitive tasks. Importantly, these differences were not explained by audibility alone. Although the cross-sectional design limits causal interpretation, the present findings suggest that hearing aid experience is associated with changes in cortical activation as WM load demands increase. Within the context of healthy aging, these findings suggest that hearing aid use is likely associated with differences in how neural networks respond to increasing cognitive demands that support everyday listening.

### Methods

#### Participants

Sixty-five participants between the ages of 50 and 90 years participated in the study. Mean (SD) age was 72.61 (7.60) years for typical-hearing adults, 74.41 (8.76) years for experienced users, and 77.60 (7.22) years for new users. Participants were recruited from Cache County, Utah, through flyers distributed in the community, word of mouth, and the campus audiology clinic. All participants were fluent in English, right-hand dominant (score > 61 on the Edinburgh Handedness Inventory)^35^, and demonstrated cognitive abilities within the normal range for their age, with Montreal Cognitive Assessment (MoCA) scores ≥ 26 ^36^.

Three groups were formed: (1) Typical-hearing adults (typical-hearing) had a speech-frequency pure-tone average (PTA; 1000, 2000, and 4000 Hz) < 30 dB HL bilaterally. (2) Experienced hearing-aid users (experienced users) had bilateral sensorineural hearing loss (PTA > 40 dB HL but not greater than 70 dB HL), with interaural PTA asymmetries not exceeding 20 db HL, and had worn programmable hearing aids for at least 6 months. (3) Newly fitted hearing-aid users (new users) met the same audiological criteria as the experienced group but were newly fitted with programmable hearing aids at the time of enrollment. Ten participants were fitted with Oticon More 1 miniRITE R hearing aids, and 10 participants were fitted with Signia C&G 7AX hearing aids, which may vary in signal processing strategies, compression, and noise management features. Experienced users had worn hearing aids for an average of 6.8 years (range = 10 mos- 25 years). In contrast, participants in the new-user group had not worn hearing aids prior to the study participation despite reporting hearing difficulties for an average of 11.5 years (range = 1–50 years). This pattern suggests that experienced users may have adopted hearing aids earlier in the course of perceived hearing difficulties; however, these estimates were based on self-reports and should be interpreted cautiously.

Demographic differences between groups were evaluated using a one-way ANOVA with Tukey post hoc comparisons, as shown in Table 4. Descriptive statistics are provided in Supplementary Table S5. No significant differences were observed for age or years of education. As expected, both hearing-aid groups had higher PTA thresholds than the typical-hearing group, while experienced and new users did not differ in PTA. Significant group differences were also observed for SII, with both hearing-aid groups showing lower SII than typical-hearing and experienced users showing lower SII than new users. A small group difference was observed in MoCA scores (Table 4); post hoc tests indicated that experienced users scored slightly higher than new users, although all groups scored within the normal cognitive range. A chi-square analysis indicated no significant differences in socioeconomic status across groups. Examination of response distributions showed that participants in all groups most frequently selected the upper-middle class category. Although the experienced-user group demonstrated a slightly broader distribution, including greater representation in lower categories, these differences were small in magnitude and should be interpreted cautiously, given low expected cell counts (see Supplementary Table S6).

All participants agreed to a comprehensive audiological assessment as part of the study. New users were compensated with a $50 Amazon gift card upon completion of the initial session and at the completion of the study, as they were enrolled in a longitudinal protocol requiring multiple visits. Experienced users were compensated with a $90 Amazon gift card due to the longer testing session, and typical-hearing adults were compensated with a $60 gift card upon completion of the study. Participants who were excluded following the cognitive assessment and comprehensive audiological evaluation were compensated $30. The study was approved by the Utah State University Institutional Review Board for research ethics and human participants. All methods were performed in accordance with the Declaration of Helsinki and the relevant guidelines and regulations of the Utah State University Institutional Review Board. Written informed consent was obtained from all participants prior to participation.

### Audiological assessment

A comprehensive audiological evaluation was conducted prior to fNIRS testing. This included otoscopic examination to ensure there was not excessive cerumen or other abnormalities of the outer ear that may interfere with testing; tympanometry using an Interacoustics Titan handheld tympanometer to verify normal middle ear status and function. Pure-tone audiometry using the Modified-Hughson Westlake procedure was completed using a GSI Suite Audiostar Pro audiometer and testing air conduction thresholds with ER-3 C insert earphones and bone conduction thresholds using a Radioear B-81 bone oscillator to determine thresholds and type of loss, ensuring that participants had sensorineural hearing loss. All participants who had conductive, mixed, or asymmetrical hearing loss were excluded from the study. Once participants were tested, the new users were fitted with Oticon More 1 miniRITE R and Signia C&G 7AX using the Verifit 2 NAL-NL2 prescriptive fitting formula, ensuring that participants met targets within 5 dB SPL at 55 dB SPL-soft, 65 dB SPL-moderate, 75 dB SPL-loud sounds, and maximum power output (MPO) to ensure soft sounds were audible and loud sounds were comfortable. Automatic adaptation was enabled over a 1-month period from level 1 to level 3 to ensure participants could adapt to the hearing aids. Datalogging features in the devices were accessed to document average daily hearing aid use. Experienced users also completed real-ear measures with the Verifit 2 using their current fitting settings to verify their hearing aids were programmed appropriately for their hearing loss. The Speech Intelligibility Index (SII) was calculated in accordance with the American National Standards Institute standard ANSI S3.5. For the experienced and new hearing-aid groups, aided SII at 65 dB values were obtained using the Audioscan Verifit2 system and used for analysis. For the typical-hearing group, unaided SII values were derived from audiometric thresholds obtained with the GSI Audiostar Pro audiometer.

**Table 4:** Participant demographics by group.

Demographics	Typical-hearing (*n* = 23)	Experienced users (*n* = 17)	New users (*n* = 20)	F(2,57)	*P*
Sex-Male	9 (39.1%)	10 (58.82%)	9 (45.0%)		
Sex-Female	14 (60.9%)	7 (41.18%)	11(55.0%)		
Age (years)	72.61 (7.60)	74.41 (8.76)	77.60 (7.22)	2.20	0.12
Education (years)	17.48 (2.57)	16.59 (2.85)	15.75 (3.08)	2.00	0.144
PTA (dB HL)	23.15 (8.54)	50.00 (7.08)	50.08 (7.05)	91.92	< 0.001
SII	80.72 (13.00)	48.65 (11.11)	61.20 (11.09)	37.17	< 0.001
MoCA	27.74 (1.45)	28.06 (1.14)	27.00 (1.21)	3.35	0.042

Values represent means and standard deviations unless otherwise indicated. Group differences for continuous variables were assessed using one-way ANOVA with Tukey post-hoc comparisons PTA = pure-tone average; SII = Speech Intelligibility Index.

### N-back Task

**Fig. 4:**
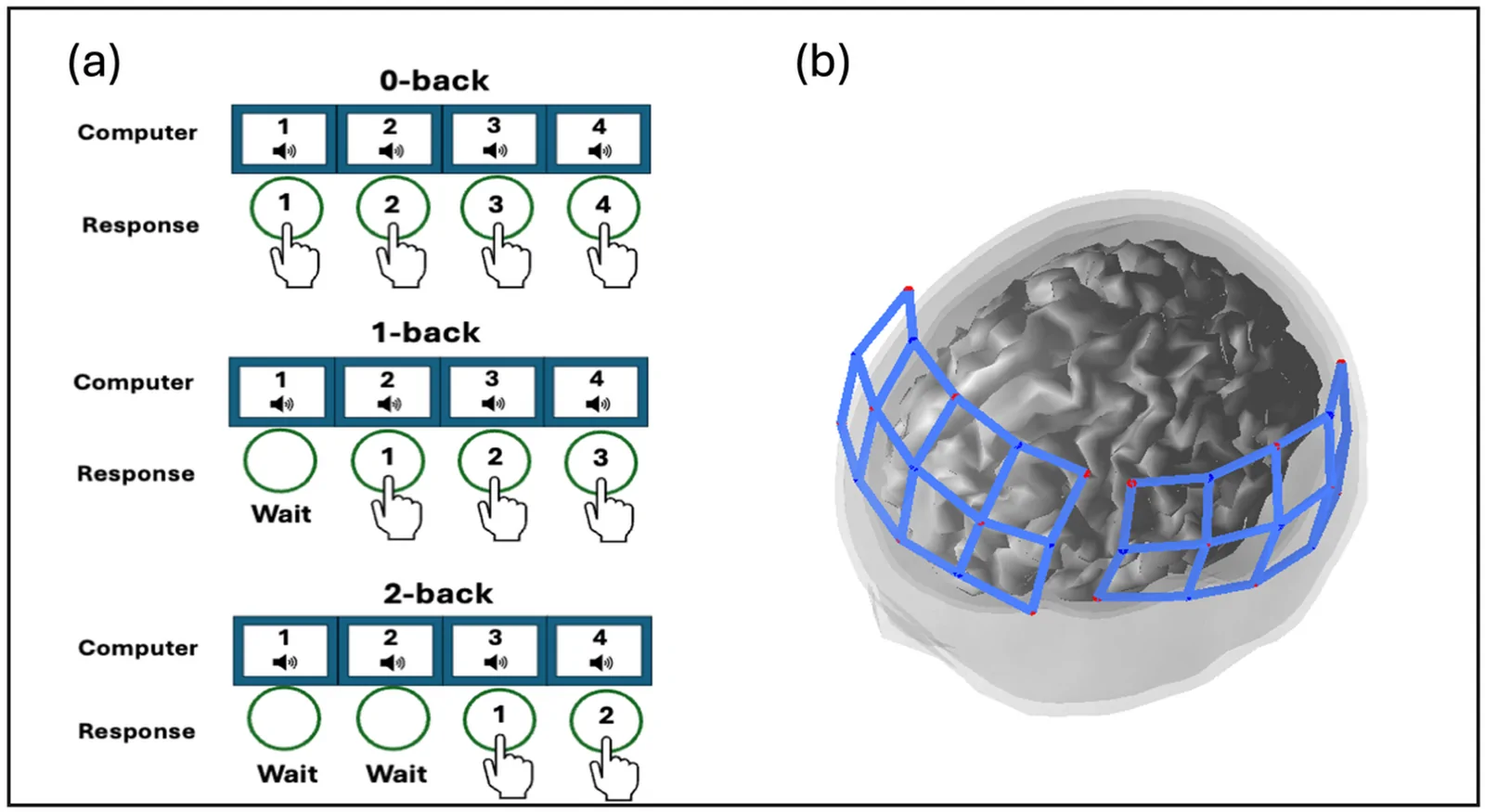
Auditory N-back and fNIRS probe arrangement. (**a**) Schematic of the auditory N-back task. Participants heard a continuous stream of spoken digits and responded by a button press. For the 1-back and 2-back blocks, participants withheld responses during the first one and two trials, so the subsequent responses could be based on the digit presented one or two trials earlier. Participants then continued to respond on each of the remaining trials. (**b**) fNIRS optode montage, illustrate the two 3 × 5 probe sets positioned to cover left dorsolateral prefrontal cortex (left DLPFC), left superior temporal gyrus (STG), and left inferior parietal lobule (IPL).

Participants completed an auditory N-back task consisting of three blocks: 0-back, 1-back, and 2-back. The N-back task had a continuous stream of spoken numbers (“1”, “2”, “3”, and “4”). The audio was played at 50 dB HL, which corresponds to a typical normal conversational level. Auditory stimuli were presented via a loudspeaker, connected to a calibrated clinical audiometer, positioned approximately 1.5 m in front of the participant. To minimize head movement and ensure consistent placement, participants rested their heads on a padded chinrest throughout the task. During the N-back task, experienced users and new users wore their hearing aids to ensure audibility of spoken numbers, and care was taken to ensure that the hearing aids did not interfere with the placement or function of the fNIRS optodes. Before beginning the task, participants completed a brief practice session to confirm that they could clearly hear and distinguish each digit. In the 0-back block, participants were instructed to press the key corresponding to the number they heard on each trial. In the 1-back block, participants pressed the key corresponding to the number presented one item prior to the current trial. In the 2-back block, participants responded when the number they heard matched the one presented two items before the current trial (see Fig. 4a). The task was adapted from^15^.

Each block contained 31 trials, each with a fixed 3-second duration. On each trial, participants heard the auditory stimulus and had up to 3 s to respond before the onset of the next trial. A 120- second fixation period preceded each block, during which participants were instructed to “look at the cross until it goes away.” The blocks were pseudorandomized and lasted 120 s total. The experiment was programmed in E-prime 3.0 (Psychology Software Tools, Pittsburgh, PA), and responses were recorded using the chronos response box, which had buttons labeled with the numbers “1”, “2”, “3”, and “4”. Dependent variables were task accuracy and fNIRS beta values at each channel.

### Procedures

Participants first underwent a training period in which a researcher read the instructions and provided an opportunity to practice each task before the actual experiment began. Before each block, participants received a reminder of the task instructions. Once participants demonstrated understanding and responded consistently during practice, researchers placed the fNIRS cap on their heads.

fNIRS data were acquired at 697.8 nm and 827.9 nm using the Hitachi ETG-4000 system (Hitachi Medical Group, Tokyo) at 10 Hz. Two 3 × 5 probe sets containing a total of 44 channels were used for data collection. One probe set was aligned with the nasion to ensure coverage of the left and right DLPFC. The second probe was placed on the left side, with the middle detector in the lowest row aligned with T3/T4, ensuring coverage of the left temporal lobe. Regions of interest (ROI) were determined a priori based on previous N-back literature and brain regions involved in auditory processing. Specifically, the ROIs included the left DLPFC (Brodmann 9/46), left IPL (Brodmann 39/40), and left STG (Brodmann 22). After the experiment was complete, researchers removed the optodes and obtained head measurements and channel locations via Polhemus PATRIOT 3D digitizer. Channel locations were registered to the Colin27 atlas^37^(see Fig. 4b).

### FNIRS processing

The NIRS Brain AnalyzIR toolbox^38^ was used for data preprocessing and channel registration. Channels were visually inspected prior to analysis to examine overall data quality. Raw intensity light values were converted to optical density values, which were then corrected for motion using the temporal derivative distribution repair procedure^39^. The corrected values were then converted to oxygenated and deoxygenated concentration values using the modified Beer-Lambert law^40^. Channel quality was evaluated using the qt-nirs toolbox, which assesses signal quality using scalp coupling index (SCI) and the peak spectral power (PSP) (SCI threshold = 0.4, *Q* threshold = 0.70, and PSP threshold = 0.05; https://github.com/lpollonini/qt-nirs). Principal component analysis was applied to the concentration values to reduce the influence of the shared global physiological signal^41^. PCA was performed across channels, and the component accounting for the largest proportion of variance shared across channels was interpreted as reflecting systemic physiology and removed prior to further analysis.

Oxygenated hemoglobin (Oxy-Hb) values were used as dependent variables in the analyses, as they exhibit a stronger response to changes in cerebral blood flow than deoxygenated hemoglobin values^42^. Due to the unique statistical properties of fNIRS, such as high serial correlation errors and heavy-tailed noise distribution, an autoregressive, iteratively reweighted least square model (prewhitening) was used. This approach provides greater statistical robustness compared to standard motion correction techniques^43,44^. Beta values were computed by solving a generalized linear model (GLM) for each channel, participant, and task. The known onsets and durations of each trial were used to define each regressor, which was then convolved with the canonical hemodynamic response function.

### Data analysis

To examine pairwise relationships between descriptive variables, pairwise scatter plots and correlation measures were calculated for age, SII, MOCA, and task accuracy (0-back, 1-back, and 2-back), broken down by group. Demographic comparisons across groups were evaluated using one-way ANOVA with Tukey post hoc tests. We modeled accuracy using a generalized linear mixed-effects model with a binomial distribution and logit link. Fixed effects included group (typical-hearing, experienced users, and new users), task (1-back, 2-back), their interaction, and age. The typical-hearing group served as the reference level for group comparisons, and the 0-back condition served as the reference level for task comparisons. Random intercepts for participant and trial were included to account for repeated measures. Interaction effects were evaluated using likelihood ratio tests. To improve model convergence, the bobyqa optimizer was used with an increased iteration limit (maxfun = 200,000).

We were also interested in determining whether experienced users with ARHL loss recruited auditory and working memory regions in a manner comparable to typical-hearing adults, and whether their activation patterns differed from those of new users. To test this, we ran mixed-effects regression models with Oxy-Hb values as the dependent measure, focusing on three regions (STG, IPL, and left DLPFC) during the auditory N-back task (1-back and 2-back). Typical-hearing adults served as the reference for group comparisons, and 1-back served as the reference for task comparisons, with 0-back speech intelligibility index (SII) and task accuracy included in the models as covariates. Oxy-Hb responses during the 0-back task were included as a covariate to account for baseline activation differences and sustained attention across groups^15^. Finally, random effects were included for each channel and participant.

All mixed-effects analyses were conducted in R (v4.2.2)^45^ using the lme4 (v1.1-19)^46^, lmerTest^47^, and emmeans^48^. Model selection proceeded hierarchically, beginning with a simple fixed-effects structure and progressively adding predictors and interactions. Model fit was evaluated using likelihood ratio tests and information criteria (AIC and BIC). Statistical significance for fixed effects was evaluated using Type III F tests with Satterthwaite approximation for degrees of freedom^49^. A Bonferroni correction was applied across the three region-of-interest models (STG, IPL, left DLPFC), with an adjusted significance threshold of α = 0.0167.

## Supplementary Information

Below is the link to the electronic supplementary material.

Supplementary Material 1

## Data Availability

Anonymized study data will be made publicly available in an open-science repository following completion of all studies within this project.

## Code availability

The code used for the fNIRS analyses in the current study will be made publicly available in an open-science repository following completion of all studies within this project.
