# Supplementary material for: Cortical activation during auditory working memory varies with hearing aid use status in age-related hearing loss
Source: Sci Rep. 2026 Jun 23;16:28672. doi: 10.1038/s41598-026-59089-3 (PMC13575199; doi:10.1038/s41598-026-59089-3)
Supplement: Supplementary file 1 [file 41598_2026_59089_MOESM1_ESM.docx]

**Supplementary Materials**

**Supplementary Table S1.** Generalized linear mixed-effects model predicting accuracy as a function of Task, Group, and Age.

| **Term** | **b** | **SE** | **z** | **p** |
| --- | --- | --- | --- | --- |
| Intercept | 10.144 | 1.780 | 5.698 | < .001 |
| 1-back | -2.686 | 0.344 | -7.801 | < .001 |
| 2-back | -4.520 | 0.341 | -13.252 | < .001 |
| Experienced users | -1.519 | 0.570 | -2.665 | 0.008 |
| New users | -3.992 | 0.537 | -7.433 | < .001 |
| Age | -0.071 | 0.024 | -2.998 | 0.003 |
| 1-back × Experienced users | 0.993 | 0.403 | 2.467 | 0.014 |
| 2-back × Experienced users | 1.331 | 0.404 | 3.297 | < .001 |
| 1-back × New users | 1.711 | 0.370 | 4.623 | < .001 |
| 2-back × New users | 2.195 | 0.372 | 5.892 | < .001 |

| **Statistic** | **Value** |
| --- | --- |
| AIC | 4557.100 |
| BIC | 4636.300 |
| Log-likelihood | -2266.600 |
| Number of observations | 5426 |
| Number of participants | 60 |
| Number of trials | 32 |
| Variance: participant intercept | 1.679 |
| Variance: trial intercept | 0.056 |

**Note.** Accuracy was modeled using a generalized linear mixed-effects model with a binomial link. Random intercepts were included for participant and trial. Coefficients are unstandardized log-odds estimates.

**Supplementary Table S2.** Simple slopes and pairwise comparisons for Group × Load interaction in STG.

| **Group** | **b** | **SE** | **95% CI** |
| --- | --- | --- | --- |
| Typical-hearing | 0.133 | 0.264 | [-0.384, 0.650] |
| Experienced users | 0.653 | 0.282 | [0.100, 1.210] |
| New users | -4.831 | 0.253 | [-5.326, -4.340] |

**Pairwise comparisons (Tukey-adjusted)**

| **Contrast** | **Estimate** | **SE** | **z** | **p** |
| --- | --- | --- | --- | --- |
| Typical-hearing vs. Experienced users | -0.521 | 0.380 | -1.369 | 0.357 |
| Typical-hearing vs. New users | 4.964 | 0.359 | 13.811 | < .001 |
| Experienced users vs. New users | 5.484 | 0.374 | 14.664 | < .001 |

**Note.** Values are averaged over Task_num and ACC. Asymptotic degrees of freedom were used. Confidence intervals are 95%.

**Supplementary Table S3.** Simple slopes and pairwise comparisons for Group × Load interaction in IPL.

| **Group** | **b** | **SE** | **95% CI** |
| --- | --- | --- | --- |
| Typical-hearing | -1.270 | 0.374 | [-2.000, -0.537] |
| Experienced users | -1.230 | 0.401 | [-2.020, -0.447] |
| New users | -3.490 | 0.364 | [-4.200, -2.778] |

**Pairwise comparisons (Tukey-adjusted)**

| **Contrast** | **Estimate** | **SE** | **z** | **p** |
| --- | --- | --- | --- | --- |
| Typical-hearing vs. Experienced users | -0.038 | 0.540 | -0.070 | 0.997 |
| Typical-hearing vs. New users | 2.220 | 0.514 | 4.322 | < .001 |
| Experienced users vs. New users | 2.258 | 0.535 | 4.220 | < .001 |

**Note.** Values are averaged over Task_num and ACC. Asymptotic degrees of freedom were used. Confidence intervals are 95%.

**Supplementary Table S4.** Simple slopes and pairwise comparisons for Group × Load interaction in left DLPFC

| **Group** | **b** | **SE** | **95% CI** |
| --- | --- | --- | --- |
| Typical-hearing | 0.379 | 0.363 | [-0.333, 1.090] |
| Experienced users | 0.663 | 0.368 | [-0.059, 1.380] |
| New users | -2.330 | 0.324 | [-2.966, -1.690] |
|  |  |  |  |

**Pairwise comparisons (Tukey-adjusted)**

| **Contrast** | **Estimate** | **SE** | **z** | **p** |
| --- | --- | --- | --- | --- |
| Typical-hearing vs. Experienced users | -0.284 | 0.510 | -0.557 | 0.843 |
| Typical-hearing vs. New users | 2.709 | 0.479 | 5.658 | < .001 |
| Experienced users vs. New users | 2.993 | 0.484 | 6.185 | < .001 |

**Note.** Values are averaged over Task_num and ACC. Asymptotic degrees of freedom were used. Confidence intervals are 95%.

**Supplementary Table S5.** Participant demographic and audiological characteristics by group, with pairwise Hedges’ g effect sizes.

| **Demographic** | **Typical-hearing**   **(n = 23)** | **Experienced users** **(n = 17)** | **New users** **(n = 20)** | **Hedges’ g (Typical vs**  **Experienced)** | **Hedges’ g (Typical vs New)** | **Hedges’ g (Experienced vs New)** |
| --- | --- | --- | --- | --- | --- | --- |
| Sex — Male | 9 (39.1%) | 10 (58.82%) | 9 (45.0%) |  |  |  |
| Sex — Female | 14 (60.9%) | 7 (41.18%) | 11 (55.0%) |  |  |  |
| Age (M, SD) | 72.61 (7.60) | 74.41 (8.76) | 77.60 (7.22) | g = -0.22 | g = -0.66 | g = -0.39 |
| Education, years | 17.48 (2.57) | 16.59 (2.85) | 15.75 (3.08) | g = 0.32 | g = 0.60 | g = 0.28 |
| PTA (M, SD) | 23.15 (8.54) | 50.00 (7.08) | 50.08 (7.05) | g = -3.53 | g = -3.38 | g = 0.09 |
| SII Average | 80.72 (13.00) | 48.65 (11.11) | 61.20 (11.09) | g = 2.60 | g = 1.59 | g = -1.10 |

**Supplementary Table S6.** Distribution of socioeconomic status categories by group and chi-square test results.
